# Cross-Excitation in Peripheral Sensory Ganglia Associated with Pain Transmission

**DOI:** 10.3390/toxins7082906

**Published:** 2015-08-04

**Authors:** Katsuhiro Omoto, Kotaro Maruhama, Ryuji Terayama, Yumiko Yamamoto, Osamu Matsushita, Tomosada Sugimoto, Keiji Oguma, Yoshizo Matsuka

**Affiliations:** 1 Department of Stomatognathic Function and Occlusal Reconstruction, Institute of Biomedical Sciences, Tokushima University Graduate School, 3-18-15 Kuramoto-cho, Tokushima 770-8504, Japan; E-Mail: c301351011@tokushima-u.ac.jp; 2 Department of Oral Function and Anatomy, Okayama University Graduate School of Medicine, Dentistry and Pharmaceutical Sciences, and Advanced Research Center for Oral and Craniofacial Sciences, Okayama University Dental School, 2-5-1 Shikata-cho, Kita-ku, Okayama 700-8525, Japan; E-Mails: maruhama@okayama-u.ac.jp (K.M.); ryujit@md.okayama-u.ac.jp (R.T.); tsugi@md.okayama-u.ac.jp (T.S.); 3 Department of Bacteriology, Okayama University Graduate School of Medicine, Dentistry and Pharmaceutical Sciences, 2-5-1 Shikata-cho, Kita-ku, Okayama 700-8525, Japan; E-Mails: yumiya@md.okayama-u.ac.jp (Y.Y.); osamu@okayama-u.ac.jp (O.M.); kuma@md.okayama-u.ac.jp (K.O.)

**Keywords:** botulinum toxin, mechanical allodynia, dorsal root ganglion, transmitter release

## Abstract

Despite the absence of synaptic contacts, cross-excitation of neurons in sensory ganglia during signal transmission is considered to be chemically mediated and appears increased in chronic pain states. In this study, we modulated neurotransmitter release in sensory neurons by direct application of type A botulinum neurotoxin (BoNT/A) to sensory ganglia in an animal model of neuropathic pain and evaluated the effect of this treatment on nocifensive. Unilateral sciatic nerve entrapment (SNE) reduced the ipsilateral hindpaw withdrawal threshold to mechanical stimulation and reduced hindpaw withdrawal latency to thermal stimulation. Direct application of BoNT/A to the ipsilateral L4 dorsal root ganglion (DRG) was localized in the cell bodies of the DRG and reversed the SNE-induced decreases in withdrawal thresholds within 2 days of BoNT/A administration. Results from this study suggest that neurotransmitter release within sensory ganglia is involved in the regulation of pain-related signal transmission.

## 1. Introduction

Peripheral nerve injury induces neuropathic pain states and hyperexcitability of neurons within sensory ganglia [1]. Despite the absence of synaptic contacts in adult sensory ganglia [2,3], the somata of sensory neurons can be transiently depolarized and cross-excited by activation of neighboring neurons within the same ganglion [4]. The prevalence of cross-excitation is increased after axotomy and might contribute to neuropathic sensory abnormalities, including pain, in patients with nerve injury [5]. Cross-excitation has been reported to be chemically mediated [6], but the precise identity of the chemical mediator is unknown. We and others previously demonstrated that neurotransmitters, such as substance P, calcitonin gene-related peptide (CGRP), and adenosine triphosphate (ATP), are released from the somata of neurons within sensory ganglia *in vivo* and *in vitro* [7,8,9,10,11], and that substance P [12] and ATP [13] excite neurons within sensory ganglia. We also showed that ganglionic neurotransmitter release increases in inflammatory and neuropathic pain states [8,14]. This suggests that neuropathy-induced increases in transmitter release from neuronal somata within sensory ganglia may result in increased cross-excitation of neighboring neurons, thereby increasing transmission of pain-related signals. If so, then blockade of neurotransmitter release within sensory ganglia should reduce neuropathic pain symptoms.

Botulinum neurotoxin (BoNT) is reported to block vesicular neurotransmitter release by disabling the soluble *N*-ethylmaleimide sensitive factor attachment protein receptor complex proteins that mediate vesicular transmitter release [15,16]. BoNT action involves three steps: extracellular binding and internalization, membrane translocation and intracellular substrate cleavage, and blockage of neurotransmitter release. The heavy chain (Hc) of BoNT plays a dual role in the BoNT toxic action of cell surface binding (binding domain; H_C_) and translocation across membranes (translocation domain, while the light chain is responsible for intracellular toxic activity. Previous studies demonstrated that BoNT is capable of attenuating the release of substance P, CGRP, and glutamate from sensory neurons in culture [17,18,19,20], in isolated preparations [21], and *in vivo* [22]. In the present study, we sought to modulate neurotransmitter release by direct application of BoNT/A to sensory ganglia in an animal model of neuropathic pain and evaluated the effect of this treatment on pain symptoms.

## 2. Results

### 2.1. Hindpaw Withdrawal Thresholds after Sciatic Nerve Entrapment (SNE) Surgery and BoNT/A Application

**Figure 1:**
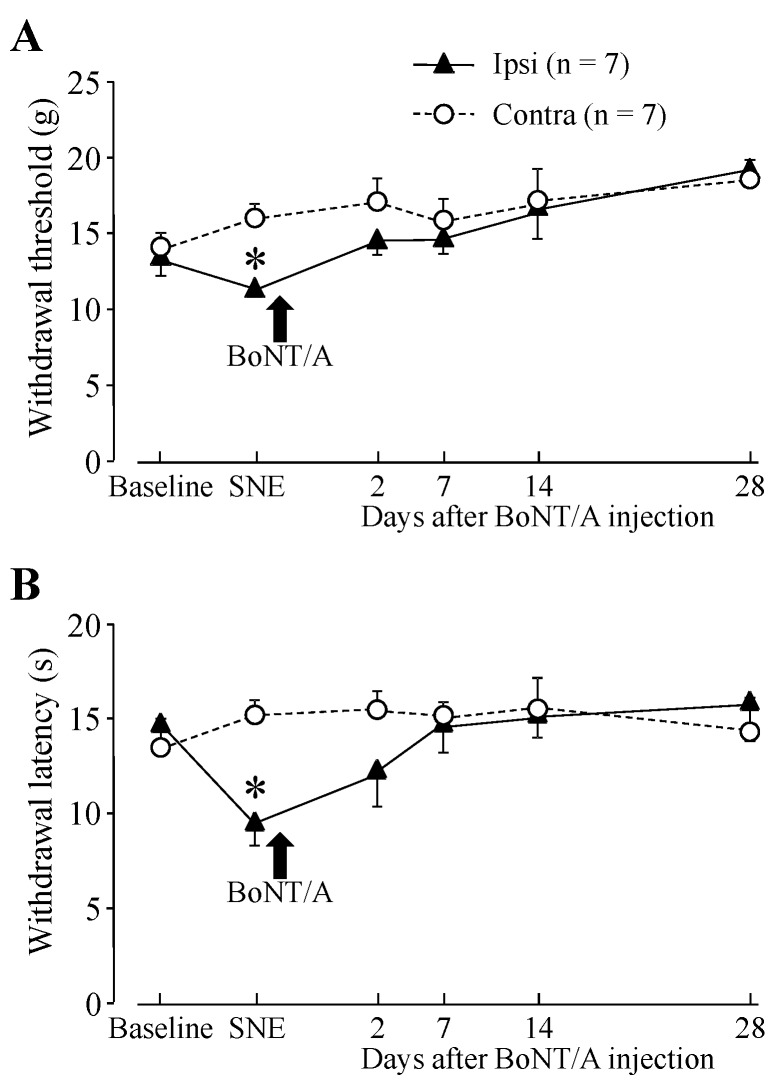
Hindpaw withdrawal thresholds after sciatic nerve entrapment (SNE) and direct botulinum neurotoxin (BoNT/A) application onto the L4 dorsal root ganglion (DRG). (**A**) mechanical stimulation; (**B**) thermal stimulation. Data are presented as mean ± S.E.M. of withdrawal thresholds to mechanical and thermal stimuli. Each group had seven animals. SNE reduced the withdrawal threshold, and direct BoNT/A application (14 s after SNE) to the L4 DRG (100 pg in 100 µL artificial cerebrospinal fluid (ACSF)) restored the threshold to the baseline level for both mechanical and thermal stimuli. * *p* < 0.05 statistical difference compared with baseline (two-way RM ANOVA with paired *t*-test).

Before cannula implantation for direct application to L4 dorsal root ganglion (DRG) and sciatic nerve entrapment (SNE) surgery, baseline measurements were obtained for both thermal and mechanical stimuli. Following SNE surgery, animals exhibited an increased sensitivity of the ipsilateral hindpaw to both types of stimuli (Figure 1). The baseline threshold for withdrawal from mechanical stimuli was approximately 14 g before SNE surgery. The threshold for the ipsilateral side significantly dropped to 11.4 g after SNE surgery (Figure 1A). The baseline latency of hindpaw withdrawal from a thermal stimulus was approximately 14 s prior to SNE surgery. Following SNE surgery (7 days), the latency significantly decreased to 9.6 s (Figure 1B). There were also signs of spontaneous pain behaviors, which included guarding behavior and changes in posture of the affected hindpaw, such as plantar flexion and toe-clenching, which are typical of this model [23]. SNE sham surgery (exposure of the sciatic nerve without touching the nerve) did not result in decreased hindpaw withdrawal threshold and latency.

**Figure 2:**
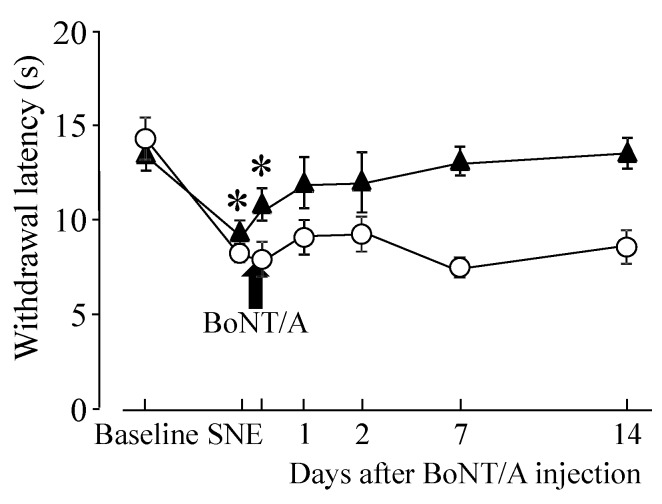
Hindpaw withdrawal thresholds with heat stimulation after SNE and/or direct BoNT/A application onto the L4 DRG. (**▲**) short duration after BoNT/A application (*n* = 7); (**○**) without BoNT/A application (*n* = 4). Data are presented as mean ± S.E.M. of withdrawal thresholds to thermal stimulation. SNE reduced the withdrawal threshold, and direct BoNT/A application (14 days after the SNE) to the L4 DRG (100 pg in 100 µL ACSF) restored the threshold to the baseline level for both mechanical and thermal stimuli. * *p* < 0.05 statistical difference compared with baseline (two-way RM ANOVA with paired *t*-test).

Direct BoNT/A application to the L4 DRG restored the threshold to the baseline level for mechanical and thermal stimulation (Figure 1). The BoNT/A effect was significant at 2 days after application and persisted for the remaining 28 days of this study. We wanted to test how quickly the BoNT/A took effect after application; therefore, we measured hindpaw withdrawal latency with heat stimulation at 2 h and 1, 2, 7, and 14 days after application. The data showed that there was some effect at 2 h after BoNT/A application (without statistical significance), and there was a statistical difference at 1 day after application (Figure 2). Figure 2 also shows that hindpaw withdrawal latency did not recover without BoNT/A application and confirmed the BoNT/A effect. BoNT/A application to the L4 DRG of sham surgery animals did not significantly change hindpaw withdrawal thresholds compared with the baseline (data not shown).

### 2.2. Motor Function after BoNT/A Application

**Figure 3:**
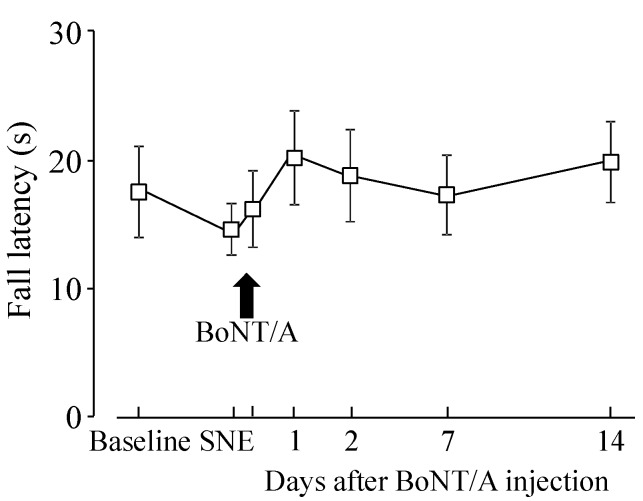
Fall latency from rotarod with SNE and direct BoNT/A application onto the L4 DRG. Data are presented as mean ± S.E.M. (*n* = 7). SNE reduced the fall latency and direct BoNT/A application (14 days after SNE) to the L4 DRG (100 pg in 100 µL ACSF) increased latency, but there was no statistical difference.

Data from the rotarod test showed that there was no statistical difference on fall latency before and after BoNT/A application (Figure 3).

### 2.3. Localization of Directly Administered BoNT/A-H_C_ in the L4 DRG

**Figure 4:**
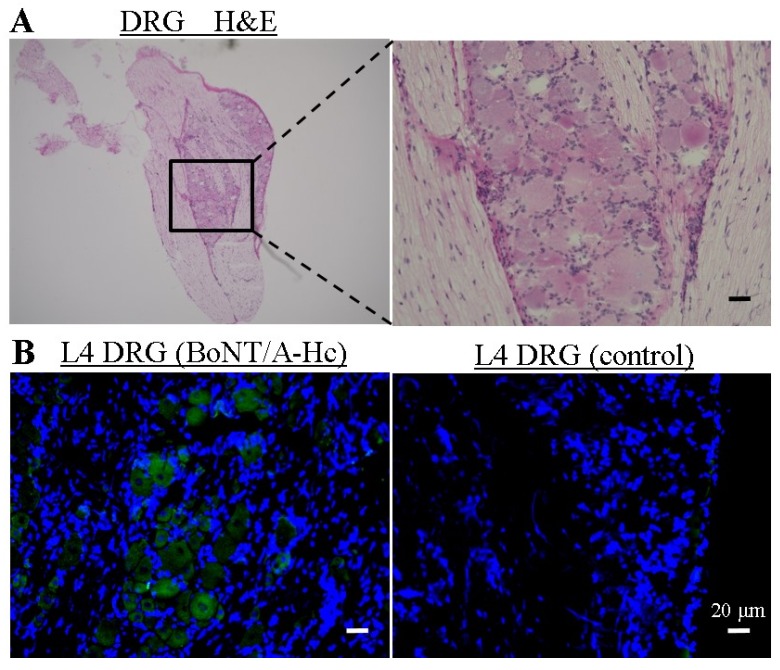
Localization of BoNT/A-H_C_ labeling with Alexa Fluor 488 L4 DRG. (**A**) Frozen sections from L4 DRG were stained with H & E; (**B**) fluorescence micrograph showing Alexa Fluor 488 (green) linked to BoNT/A-H_C_ and co-localization with DAPI-stained nucleus (blue). As a control, the solvent liquid with Alexa Fluor 488 was administered one hour before extraction. BoNT/A-Hc was taken up into the L4 DRG. The scale bar is 20 µm.

We examined the localization of directly administered the half C-terminal of heavy chain of BoNT/A (BoNT/A-H_C_) in the L4 DRG in order to confirm its site of action indirectly. Frozen sections were stained with hematoxylin and eosin (H & E) (Figure 4A). We observed that cells in the DRG had a centrally placed nucleus and satellite cells surrounded the DRG cells. Furthermore, fluorescent sections had the morphological similarities to H & E sections. BoNT/A-H_C_ was labeled with Thiol-Reactive Probes (Alexa Fluor 488), which was taken up into the BoNT/A-H_C_ injection site in the L4 DRG, not into the control site injected the solvent liquid with Alexa Fluor 488 (Figure 4B).

## 3. Discussion

The main findings of this study were as follows: (i) SNE decreased the hindpaw withdrawal threshold to mechanical stimulation and withdrawal latency to thermal stimulation, and direct application of BoNT/A to the L4 DRG counteracted these effects; (ii) direct application of BoNT/A-H_C_ was localized in the cell bodies of the L4 DRG; (iii) direct application of BoNT/A to the L4 DRG did not affect motor function.

Peripheral nerve injury increases excitability of primary sensory neurons; this increase is widely considered to contribute to the behavioral symptoms of neuropathic pain [24,25]. The demonstrated increases in neurotransmitter release from hyperexcitable afferents within the dorsal horn [19,26,27,28] and at the peripheral injury sites [29] are also thought to contribute to neuropathic pain, particularly to peripheral and central sensitization. Previous studies demonstrated that intrathecal administration of BoNT/A or BoNT/B cleaved SNAP-25 in the spinal cord and urinary bladder, decreased neurotransmitter release from spinal afferents, decreased pain behavior, and did not affect motor function [30,31]. These studies showed that blocking neurotransmitter release in the spinal cord decreases pain information transmission without motor function impairment.

Peripheral administration of BoNT/A induces cleavage of SNAP-25 in peripheral nerve endings, DRG, and the spinal cord [32]. This study showed that BoNT/A cleaves SNAP-25 in DRG, even though there are no synapses in DRG. Other studies have demonstrated that BoNT administered to the innervation area of injured sensory neurons is capable of attenuating the release of substance P, bradykinin, CGRP, and glutamate from DRG and trigeminal ganglion neurons, as well as alleviating nocifensive behaviors [20,33,34]. Subcutaneous injections of BoNT in the receptive fields of primary afferent neurons are expected to decrease exocytotic release of chemical substances at the peripheral terminals of these sensitized afferent fibers, thereby alleviating pain symptoms. However, there is also evidence that subcutaneous BoNT/A injections in the peripheral region result in retrograde transport in axons projecting away from the injection site, eventually affecting SNAP-25 cleavage and transmitter release within the somata of sensory ganglion neurons and possibly at their central terminals [32,34]. Studies have also demonstrated the ability of retrogradely transported BoNT/A to undergo transcytosis [35].

One concern with subcutaneous injections of BoNT is that they may also affect nearby muscle contractility, which could contribute to the apparent alleviation of pain symptoms. Furthermore, these studies did not directly address the possible contribution of intra-ganglionic transmitter release to pain symptoms. Here, we administered BoNT/A directly to the sensory neurons within the L4 DRG to directly affect ganglionic transmitter release, thereby avoiding possible confounding effects on motor contractility. 

The observed decreases in pain symptoms of SNE rats after direct ganglionic administration of BoNT/A suggest that exaggerated transmitter release within sensory ganglia is an important contributor in the maintenance of neuropathic pain. Takeda *et al.* (2005) demonstrated that temporomandibular joint inflammation alters excitability of Aβ trigeminal primary neurons that innervate the facial skin. They also showed that activation of neurokinin 1 receptors within the trigeminal ganglion increases Aβ-neuron excitability and contributes to facial mechanical allodynia after temporomandibular joint inflammation, and conversely that ganglionic blockade of NK receptors decreases Aβ-neuron excitability and alleviates facial mechanical allodynia [36]. Collectively, these studies point to the importance of increased intra-ganglionic transmitter release and cross-excitation of neighboring neurons to the development and maintenance of chronic pain states.

## 4. Materials and Methods 

All experimental procedures were carried out in accordance with the NIH guidelines on animal care, and the animal protocol was approved by Okayama University (OKU-2011190) and Tokushima University (H25-294).

All surgical procedures were performed under anesthesia with pentobarbital sodium (50 mg/kg, i.p.) and under sterile conditions.

### 4.1. BoNT/A and Fluorescent Labeling of Recombinant BoNT/A-H_C_

BoNT/A was purified by the authors [37]. To prepare the fluorescence-labeled BoNT/A-H_C_ recombinant protein, we followed previously described methods for tetanus toxin (TeNT) [38]. DNA encoding BoNT/A-H_C_ was inserted into *Sma*I and *Not*I sites of pGEX-6P-3 vector. To produce BoNT/A-H_C_ tagged to the peptide A EAAAR EACCR ECCAR EAAAR A (cysteine-rich tag), two complementary oligonucleotides, 5′-GATCCGCAGAGGCAGCAGCACGAGAGGCTTGTTGTCG AGAGTGTTGTGCACGAGAGGCAGCAGCACGAGCCC-3′ and 5′-GGGCTCGTGCTGCTGCCT CTCGTGCACAACACTCTCGACAACAAGCCTCTCGTGCTGCTGCCTCTGCG-3′, were annealed, and then ligated into *Bam*HI and *Sma*I sites of the pGEX-6P-3-BoNT/A-H_C_. The resulting plasmid (pGEX-6P-3-Cys-BoNT/A-H_C_) was introduced into competent *Escherichia coli* BL21. The recombinant Cys-BoNT/A-H_C_ fused to glutathione S-transferase (GST) was purified by Glutathione Sepharose 4B affinity chromatography (GE Healthcare Japan, Tokyo, Japan). The GST tag was cleaved by PreScission Protease (GE Healthcare Japan, Tokyo, Japan), and then eliminated by reapplying it to Glutathione 4B affinity column. After affinity purification and GST-tag removal, we harvested the protein solution containing the recombinant Cys-BoNT/A-H_C_. BoNT/A-H_C_ with a cysteine-rich tag at the *N*-terminus was predicted to form α-helices with cysteines favorably oriented to bind Alexa maleimides. Labeling with the Alexa Fluor 488 maleimides (Invitrogen, Carlsbad, CA, USA) was performed according to the manufacturer’s instructions.

### 4.2. Cannula Implantation in L4 DRG

Cannula implantation surgery was performed with a slight modification of previously described methods [39]. Male Sprague–Dawley rats (300–350 g) were anesthetized, and the hair in the surgical area was shaved and the skin was sterilized with povidone-iodine solution. An incision was made along the midline of the back at the L3 to L5 spinal level. Following separation of the right or left paraspinal muscles from the transverse processes, the L4 transverse process was removed to open the intervertebral foramen. A flexible polyethylene cannula (outer diameter = 1.3 mm, inner diameter = 0.9 mm; Igarashi Ika Kogyo Co., Tokyo, Japan) was attached to the L3 transverse process with cyanoacrylate adhesive, and the tip of the cannula was so placed to slightly touch the L4 DRG. The fascia and the skin were sutured (4-0 nylon; Softretch^®^, GC Co., Tokyo, Japan). The cannula was filled with artificial cerebrospinal fluid [ACSF: NaCl 119, KCl 5, glucose 30, HEPES 25, CaCl_2_ 2, MgCl_2_ 2 (mM)), and the other end of the tube (exteriorized to the back of the rat) was pinched with heated forceps to seal the opening. Fourteen days after SNE surgery, the rats were re-anesthetized with pentobarbital (50 mg/kg, i.p.) and purified BoNT/A (100 pg in 100 µL ACSF) [37] or Alexa-labeled BoNT/A-H_C_ (2 µg in 100 µL, control is a solvent liquid with Alexa-488 (Invitrogen, Carlsbad, CA, USA)) was applied to the L4 DRG by injection over ~10 s through the exteriorized end of the cannula.

### 4.3. Sciatic Nerve Entrapment 

After recording the hindpaw withdrawal threshold, the rats were anesthetized and the SNE surgical procedures were performed as previously described [23]. Cannula implantation and SNE surgeries were done on the same day. The hair on the lower back and thigh of the rats was shaved, and the skin was sterilized. A skin incision was made in one thigh, and the sciatic nerve was exposed through a blunt dissection of the overlying muscle. Three Tygon cuffs (length = 1 mm, outer diameter = 2.28 mm, inner diameter = 0.76 mm) were placed around the exposed sciatic nerve. The tubes of this size snugly fitted around the sciatic nerve without constricting it. The muscle layer was closed with absorbable sutures (5.0 Vicryl, Ethicon, Johnson & Johnson, New Brunswick, NJ, USA), and the skin was closed with a 4-0 nylon suture. In some rats, the sciatic nerve was exposed without cuff placement. Sutures were removed 7–10 days post-surgery under isoflurane anesthesia.

### 4.4. Behavioral Testing

Mechanical and thermal sensitivities were recorded in this study. Mechanical sensitivity was assessed using the electronic von Frey hair pressure transducer (Dynamic Plantar Aesthesiometer 37450, Ugo Basile, Varese, Italy). Baseline behavioral testing was performed 1–2 days before cannula implantation and SNE surgeries. Testing was repeated for 7 days post-SNE surgery and for 14 or 28 days post-BoNT/A injection. The rat was gently placed in a plastic-walled cage (10 × 20 × 13 cm) with a metal mesh floor (0.6 × 0.6 cm holes). The tip of the electronic von Frey filament was pressed (10-s ramp) onto a point in the middle of the hindpaw until the animal withdrew the foot. The force (g) applied at the time of withdrawal was recorded. Each hindpaw was tested five times at 1-min intervals, and the results were averaged for each paw for that day. Thermal sensitivity testing was performed using the Hargreaves apparatus (Plantar Test, 37370, Ugo Basile, Varese, Italy), which measures withdrawal latency from a radiant heat source directed at the proximal half of the plantar surface of each hindpaw. The baseline and post-operative testing schedule was the same as for the mechanical sensitivity test. Prior to testing, rats were allowed to acclimate for 15 min to the testing environment, which consisted of translucent plastic-walled individual chambers (10 × 20 × 13 cm) and a 3-mm thick glass bottom. A radiant heat source, consisting of an adjustable infrared lamp and a built-in stopwatch accurate to 0.1 s, was used to measure paw withdrawal latency. Each paw was tested five times at 25% maximal heat intensity (45 °C), allowing 5 min between each test. The test was performed only when the rat was stationary. Special care was taken to keep the glass bottom clean and dry during testing. If the glass required cleaning during experimentation, the rats were allowed 5–10 min to reacclimatize to the environment. Results from five tests were averaged for each paw for that day. Motor function was tested using the rotarod rest (LE8500, Bio Research Center, Nagoya, Japan) to check side effects of BoNT/A application. The rotarod test was conducted after the rats were habituated with the apparatus. The rotarod was started at a low speed (5 rpm) and the speed gradually increased from 5 to 40 rpm over 60 s. The duration from the start to the time that the rats fell down was recorded.

### 4.5. Histology Sample Preparation 

For the histological study, we used different animals than those used for behavioral testing. At 14 days after cannula implantation surgery and infusion of BoNT/A-H_C_, animals were re-anesthetized and transcardially perfused with saline followed by 4% paraformaldehyde in 0.1 M phosphate buffer (PB; pH 7.4). L4 DRGs were resected, postfixed in the same fixative for 24 h, and then immersed in 20% sucrose in 0.02 M phosphate-buffered saline (pH 7.4) for 48 h. The DRGs were embedded in Tissue-Tek (Agar Scientific, Stansted, UK) optimal cutting temperature compound and flash frozen in liquid nitrogen. Frozen sections, 10 µm thick, were cut on a cryostat and mounted onto silane-coated slides (Matsunami, Osaka, Japan).

### 4.6. Histological Assessment

DRG sections were subjected to H & E staining, and nuclei were stained with 4'-6-diamidino-2-phenylindole (DAPI) (Life Technologies, Tokyo, Japan). Two sets of sections were used, one for H & E staining and the other for DAPI staining. The latter slides were mounted with fluorescent mounting medium (DAKO, Glostrup, Denmark). Fluorescence and optical light microscopic images were obtained and analyzed with a BZ-X710 all-in-one fluorescence microscope (Keyence, Osaka, Japan).

### 4.7. Statistical Analysis

All data are presented as mean ± S.E.M. Statistical analysis was performed using one-way analysis of variance (ANOVA) or two-way repeated measures ANOVA followed by paired *t*-test with statistical software R. The criterion used for statistical significance was *p* < 0.05. The *p*-value adjustment method was Bonferroni.

## 5. Conclusions

We found that SNE decreased hindpaw withdrawal threshold to mechanical stimulation and withdrawal latency to thermal stimulation, and direct application of BoNT/A to the L4 DRG restored nocifensive parameters to near-baseline levels. These results suggest that ganglionic application of BoNT/A alleviates neuropathy-induced behavior. These findings also suggest that neurotransmitter release in sensory ganglia is involved in the regulation of pain transmission and may therefore represent an additional target for the management of chronic pain symptoms. 
